# Health and educational aspirations in adolescence: a longitudinal study in Finland

**DOI:** 10.1186/s12889-019-7824-8

**Published:** 2019-11-04

**Authors:** Henrik Dobewall, Pirjo Lindfors, Sakari Karvonen, Leena Koivusilta, Mari-Pauliina Vainikainen, Risto Hotulainen, Arja Rimpelä

**Affiliations:** 1 Faculty of Social Sciences (Health Sciences), Tampere University, Po Box 20, (Arvo Ylpön katu 34), 33014 Tampere, Finland; 2 PERLA—Tampere Centre for Childhood, Youth and Family Research, Tampere University, 33014 Tampere, Finland; 3 Social Policy Research Unit, Finnish Institute for Health and Welfare, 00271 Helsinki, Finland; 4 Department of Social Research, Faculty of Social Sciences, University of Turku, Turku, Finland; 5 Faculty of Education, Tampere University, 33014 Tampere, Finland; 6 Centre for Educational Assessment, University of Helsinki, 00014 Helsinki, Finland; 7 Department of Adolescent Psychiatry, Pitkäniemi Hospital, Tampere University Hospital, 33380 Nokia, Finland

**Keywords:** Health selection, Social causation, Adolescents, Educational trajectories, School survey, aspirations

## Abstract

**Background:**

The health selection hypothesis suggests that poor health leads to low educational attainment during the life course. Adolescence is an important period as poor health might prevent students from making the best educational choices. We test if health in adolescence is associated with educational aspirations and whether these associations persist over and above sociodemographic background and academic achievement.

**Methods:**

Using classroom surveys, a cohort of students (*n* = 5.614) from the Helsinki Metropolitan Region was followed from the 7th (12–13 years,) up to the 9th grade (15–16 years) when the choice between the academic and the vocational track is made in Finland. Health factors (Strengths and Difficulties Questionnaire (SDQ), self-rated health, daily health complaints, and long-term illness and medicine prescribed) and sociodemographic background were self-reported by the students. Students’ educational aspirations (applying for academic versus vocational track, or both) and their academic achievement were obtained from the Joint Application Registry held by the Finnish National Agency for Education. We conducted multilevel multinomial logistic regression analyses, taking into account that students are clustered within schools.

**Results:**

All studied health factors were associated with adolescents’ educational aspirations. For the SDQ, daily health complaints, and self-rated health these associations persisted over and above sociodemographic background and academic achievement. Students with better health in adolescence were more likely to apply for the academic track, and those who were less healthy were more likely to apply for the vocational track. The health in the group of those students who had applied for both educational tracks was in between. Inconsistent results were observed for long-term illness. We also found robust associations between educational aspirations and worsening health from grade 7 to grade 9.

**Conclusions:**

Our findings show that selection by health factors to different educational trajectories takes place at early teenage much before adolescents choose their educational track, thus supporting the health selection hypothesis in the creation of socioeconomic health inequalities. Our findings also show the importance of adolescence in this process. More studies are needed to reveal which measures would be effective in helping students with poor health to achieve their full educational potential.

## Introduction

Years of schooling and the level of education are associated with virtually all health outcomes: the higher educational attainment, the better health [1–4]. Two main mechanisms to explain these relationships have been presented: the social causation hypothesis and the hypothesis of health selection that can differ in importance at different periods of life course [5–7]. In this paper, our focus is the health selection in adolescence. Adolescence is a sensitive period from the point of view of future educational plans as well as for the development of health and risk factors for health [8].

Prospective cohort studies that investigate the effect of health in adolescence on educational attainment are accumulating slowly. Some studies support the selection hypothesis. Studies from Finland and the USA have shown that diverse health factors, e.g. self-rated health, psychosomatic symptoms, and long-term illness in adolescence predict later educational outcomes [6, 9–13]. Studies that controlled for unobserved person or family characteristics have shown that the education-health gradient is largely shaped by health selection in adolescence [6, 11]. Some studies have not found support for the health selection hypothesis. Depressive symptoms in adolescence were not related to life-course trajectories of education and work in a Swedish study [14], and hardly any association was found between timely graduation from secondary education and health records in a Dutch study [15]. A study from New Zealand showed that social problems but not the psychological ones were associated with later educational attainment [16]. In summary, the findings of these prospective studies testing if health in adolescence influences education at a later age are mixed. The differences can be based on different samples, studied health factors, or which educational outcomes have been used.

Also, the educational context differs between countries. We study here the process of health selection in Finland, a Nordic welfare state with a 9-year comprehensive school with a national curriculum. In grades 7 to 9 (lower secondary school) most subjects have a subject teacher while the lower grades 1 to 6 are taught by a class teacher. Compared to many other countries [17], tracking to different school paths takes place quite late, in the 9th grade (age 16) when compulsory schooling ends. Virtually all adolescents apply to upper secondary education, and do that through a national Joint Application System (https://studyinfo.fi/wp2/en/valintojen-tuki/finnish-application-system), following their educational aspirations for schools of the academic track, the vocational track, or both. The selection of students is based on their preferences and grade point average – GPA –. This makes Finland an ideal context for studying the relationship between health and educational aspirations in adolescence.

Educational aspirations are the first step in the process of the formation of one’s educational path. They are defined as abstract statements and beliefs about students’ future plans such as the level of education one wishes to achieve [18, 19]. They are a strong predictor of future educational trajectories and through that their adult socioeconomic position [18, 20, 21]. Poor health, however, might distort the development of educational aspirations and consequently prevent students from realizing their full educational potential. Health disadvantage and lower levels of education in combination might thus lead to diminished economic returns in the form of labor earnings in adulthood [22]. Only a few studies have investigated how health in adolescence is related to educational aspirations. One of the few is a Canadian study which showed that fewer adolescents with physical disabilities had plans for education after high school [23]. Another study from Slovakia showed that self-rated health was not related to educational aspirations among students in three different school tracks [24]. It is therefore currently not known which health factors might influence adolescents’ plans for further education.

Academic achievement is a strong predictor of a student’s educational trajectory, but even in a Nordic welfare state like Finland, parents’ education and employment predict their children’s academic achievement and choice of educational tracks [25–27]. In addition, other sociodemographic factors such as gender, immigrant background, and family structure are known to be associated with educational choices [24, 28, 29]. When studying the independent effect of health on educational aspirations, the sociodemographic background and the academic achievement of the student need to be controlled for.

Health selection in adolescence can be a pathway to future health inequalities. With this study, we want to generate knowledge on whether health in adolescence patterns educational aspirations and through that educational trajectories. Based on the above, we hypothesize that health in adolescence is related to educational aspirations so that students with better health are more likely to apply for the academic track and those who are less healthy, are more likely to apply for the vocational track. It is well-known that adolescents’ sociodemographic background and particularly academic achievement strongly predict educational trajectories. In accordance with the health selection hypothesis, we hypothesize, however, that adolescents’ health has an effect over and above these predictors. The research questions are: Are health factors associated with adolescents’ educational aspirations and do these associations persist over and above sociodemographic background and academic achievement? Does health matter already at the beginning of 7th grade (age 12–13 years) when students start lower secondary education or does health matter only at the end of the 9th grade (age 15–16 years) at the time when they apply to upper secondary education? Finally, we want to find out whether health improvement or worsening from the 7th to 9th grade is associated with adolescents’ plans for education after compulsory schooling.

## Methods

### Procedure and setting

The study was conducted as part of the project “Redefining adolescent learning: A multilevel longitudinal cohort study of adolescent learning, health, and well-being in educational transitions in Finland” – Metropolitan Longitudinal Finland (MetLoFin) –. It follows a large cohort of students from the Helsinki Metropolitan Region from the lower secondary education to the end of upper secondary education. In 2011, all 7th graders (12–13 years old) were invited to participate. The recruitment occurred through the educational authorities of all 14 municipalities of the Helsinki Metropolitan Region, each of which gave a permission for the study. A follow-up survey was fielded in 2014 when the students were in the 9th grade (15–16 years old).

The study protocol was approved by the Ethical Committee of the Finnish Institute for Health and Welfare. In line with the instructions of the Finnish National Board on Research Integrity (TENK) in 2009, no parental consent was required when the study was conducted as part of the students’ normal schoolwork. Two of the 14 municipalities had adopted a policy that a written parental consent is always required. These were collected. In the other municipalities, information letters were sent to the parents who had the possibility to withdraw their child from the study. The students were instructed about the purpose of the study and that participation was voluntary and that they can decline to answer any question or withdraw from the survey at any time. This was mentioned at the beginning of the questionnaire at the first pageRegistry data on students’ educational aspirations were obtained from the Finnish National Agency for Education, covering the period from Spring 2014 to Spring 2017. In Finland, students can apply via the Joint Application System to a maximum of five study places in upper secondary schools, ranked in the order in which they wish it to be selected. There are two general application rounds—Spring and Autumn—which are followed by an additional application round in which students can apply for vacant study places. Combining the survey answers with the Joint Application Registry was done by a data manager who does not analyze the data himself.

**Fig. 1:**
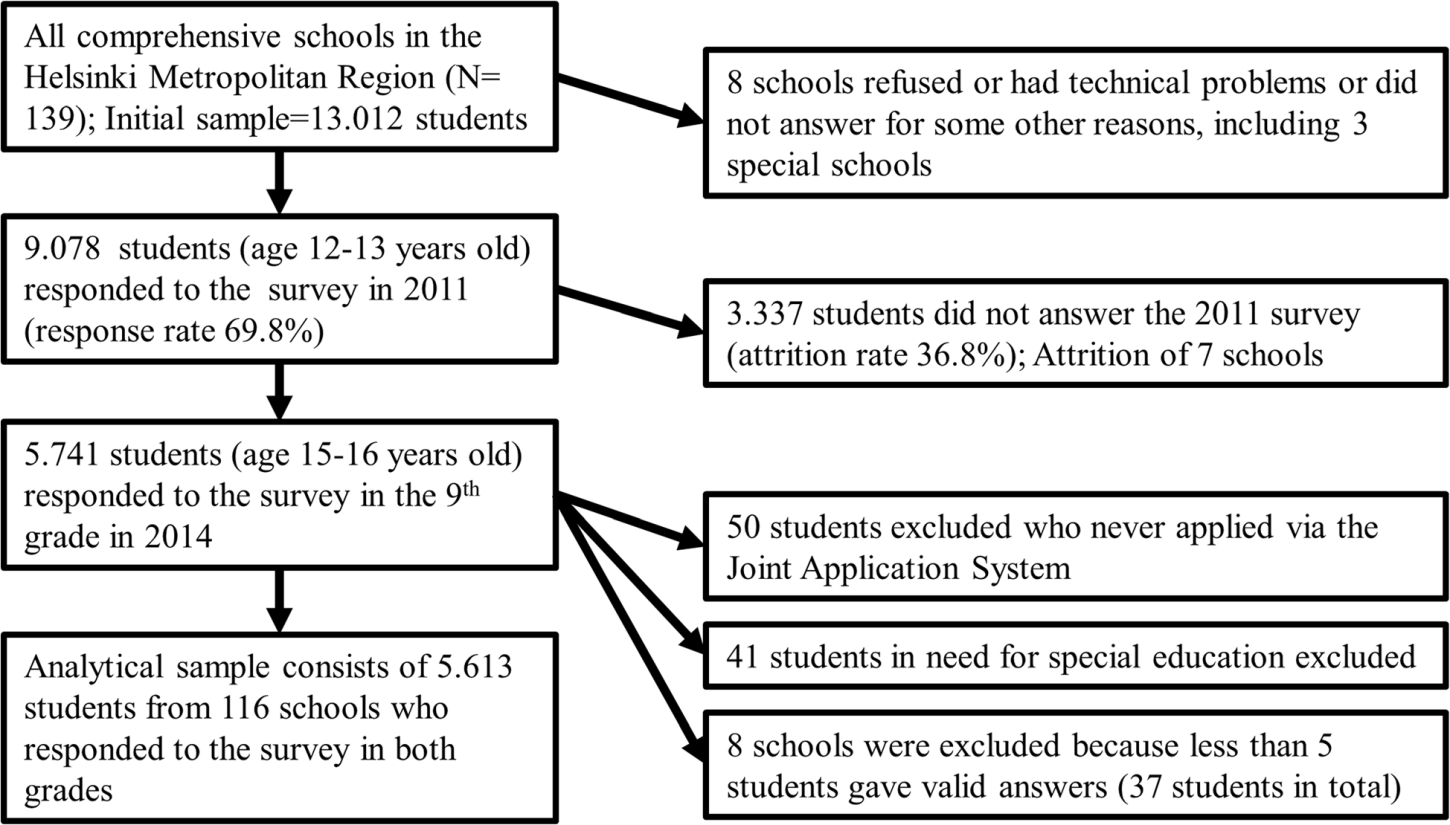
Flow diagram representing the formation of the study population. The numbers in the final analyses differ due to missing information in the predictor variables

In total, 13,012 students belong to the baseline sample of the MetLoFin project (for a flow diagram representing the formation of the study population, see Fig. 1). In total, 9.078 students (50.0% female) answered the health questionnaire in the 7th grade (response rate of 69.8%). Of these students, 5.741 also participated in the 9th grade (attrition rate of 36.8%). We excluded from the analyzes, those students who never applied via the Joint Application System (*n* = 50), who had applied for special education at some point (*n* = 41) [30], or who came from schools where less than five students gave valid answers [31]. The analyzed sample consists of the remaining 5.614 students from 116 schools who responded to both surveys and fulfilled our inclusion criteria. The numbers in the final analyses differed due to missing information in the predictor variables.

### Dependent variable: educational aspirations

The information available in the Joint Application System was used to construct an objective measure of students’ educational aspirations. The resulting variable had three categories: students who “Applied for the academic track” (58.0%, *n* = 3.258), “Applied for the vocational track” (19.8%, *n* = 1.111), or were undecided about their future plans and “Applied for both educational tracks” (22.2%, *n* = 1.244). We treated the recordings of students’ choices as educational aspirations regardless some of the students when applying for a study place did not know whether their GPA will be good enough to be selected, and some of them did not acquire any place to study. Nevertheless, these were their aspirations.

### Health factors

#### Strengths and difficulties questionnaire

The Strengths and Difficulties Questionnaire (SDQ) version suitable for adolescents was administered [32, 33]. It measures emotional symptoms, conduct, hyperactivity/inattention, and peer relationship problems with five questions each. The students marked on a 3-point fully labeled Likert scale (0 = “Not true” 1 = “Somewhat true,” 2 = “Certainly true”) which of the twenty attributes described them best over the past 6 months. The answers were summed together to generate a total difficulties score of psychosocial problems that was categorized to “Normal” (score < 13), “Slightly raised” (14–19), and “High” difficulty score (20–40). Previous work using the same data as in the current study had found good psychometric properties for the SDQ [34].

#### Daily health complaints

Daily health complaints were assessed with the frequency of ten psychosomatic symptoms (headache, neck and shoulder pain, lower back pain, stomach aches, tension and nervousness, irritability or outbursts of anger, trouble falling asleep or waking at night, feeling tired or weak, feeling dizzy, trembling of hands) experienced daily over the past 6 months [35]. Answers were provided on a 4-point fully labeled Likert scale. Students with severe health complaints nearly every day were classified as “No symptoms,” “One symptom,” and “Two or more.”

#### Long-term illness

Long-term illness was assessed with two “Yes/No” questions. The students were asked whether they had a long-term illness or disability and whether they regularly used medicine prescribed by a doctor. The answers were categorized into a single variable: “No long-term illness,” “Long-term illness,” and “Medicine prescribed.”

#### Self-rated health

Students’ subjective evaluation of their health was assessed with a single question [36]. The answers were provided on a 5-point Likert scale. The self-rated health scale was dichotomized comparing students who answered “Good” to those who answered “Average or poor.”

#### Missing values and change from grade 7 to grade 9

To report analyses that are as representative as possible, we have filled missing values in the health factors using the second or previous measurement (21–149 missing values were replaced, respectively). To assess the within-person change in health from grade 7 to grade 9, we calculated for each of the health factors a difference score [37]. The resulting variables contrasted students who remained stable with those whose health improved or worsened over time (for frequencies, see Additional file 1: Table S1).

### Background variables

#### Sociodemographic background

We used students’ *gender* to account for potential differences between “Girls” and “Boys.” We further used *parental employment* (“Both parents working” versus “Other”), *parental education* (“Low” versus “High,” that is at least one parent being highly educated with matriculation examination or university degree), *immigrant background* (Finnish−/Swedish-speaking “Natives” were compared to “Immigrants,” who had moved to Finland and/or had at least one parent who was born abroad), and *family structure* (“Nuclear family” versus “Other”) as control variables. Although already 11-year-olds were found to provide valid and detailed information about their parents’ economic activity and occupation [38], we gave preference to students’ answers to their sociodemographic background provided in the 9th grade. Only in the case of missing data, answers provided by the students in the 7th grade were used*.*

#### Academic achievement

In the Finnish education system, students both apply to the upper secondary education and are accordingly sorted into educational tracks mostly by their grade point average – GPA – which results from performance in different study subjects graded by the subject teachers. Grades from the school leaving certificate (from 9th grade) are also included in the Joint Application Registry. The GPA of each student was calculated based on his/her grade in mother tongue, foreign language, mathematics, and science (averaging grades in biology, geography, physics, and chemistry). Academic achievement (GPA) was categorized as “High” (9–10 (excellent) points), “Medium” (7.5–8.5 points), and “Low” (4 (fail) - 7 points).

### Analytical strategy

Multilevel multinomial logistic regression analyses with random effects were estimated with generalized structural equation modeling using Stata Version 15. First, we calculated the variance in educational aspirations attributable to differences between schools which the students attended in the 7th grade. Second, we regressed students’ choices between the educational tracks on their health in the 7th grade (12–13 year-olds) and repeated this analysis with students’ health in the 9th grade (15–16 year-olds). Third, we controlled for the sociodemographic background of the students. Fourth, students’ academic achievement was entered into the models. Finally, we looked at within-person changes in health factors over time. Students’ health factors in the 7th grade were included in this analysis to account for starting levels and potential ceiling effects. The results of this analysis of within-person change, however, should not be interpreted as fixed effects estimates because our outcome variable educational aspirations did not change over time [39]. In all models, we controlled for gender differences. The model parameters were presented as odds ratios (OR) with 95% confidence intervals (CI). Akaike (AIC) and Bayesian (BIC) information criteria were reported for comparing the fit of the models to the data. Interaction effects between gender and health factors were not significant (results not shown).

### Attrition analyses

An independent samples t-test revealed that students that answered the survey in both the 7th and the 9th grade were more likely to have better grades than those who dropped out (*p* < .001). Chi-squared tests revealed that, in the 7th grade, non-participants were also more likely to have psychosocial problems, long-term illness and medicine prescribed, and more daily health complaints (*p* < .001). There were also statistically significant differences in frequencies for all sociodemographic variables but gender. Participants were more likely to have highly educated and working parents (*p* < .001) and to live in a nuclear family (*p* < .05), and were less likely immigrants (*p* < .001) than non-participants.

## Results

The proportions of students in relation to the study variables are presented in Table 1 grouped by the students’ educational aspirations.

**Table 1:** Health Factors, Sociodemographic Background, and Academic Achievement: Descriptive Statistics by Educational Aspirations, % (n)

	Applied for vocational track	Applied for both tracks	Applied for academic track	Total
Strengths and Difficulties Questionnaire = SDQ, 7th grade				
Normal	78.7 (873)	80.6 (1.002)	89.1 (2.900)	85.2 (4.775)
Slightly raised	14.1 (156)	13.7 (170)	8.3 (270)	10.6 (596)
High difficulty score	7.2 (79)	5.7 (71)	2.6 (85)	4.2 (235)
Strengths and Difficulties Questionnaire = SDQ, 9th grade				
Normal	63.0 (698)	66.8 (830)	80.0 (2.603)	73.7 (4.131)
Slightly raised	19.6 (217)	19.3 (240)	13.2 (431)	15.8 (888)
High difficulty score	17.4 (193)	13.9 (173)	6.8 (221)	10.5 (587)
Daily health complaints, 7th grade				
No symptoms	73.7 (818)	73.6 (915)	79.3 (2.583)	76.9 (4.316)
One symptom	15.6 (173)	15.4 (191)	12.6 (409)	13.8 (773)
Two or more	10.7 (119)	11.1 (138)	8.2 (266)	9.3 (523)
Daily health complaints, 9th grade				
No symptoms	69.2 (768)	68.9 (857)	73.2 (2.386)	71.5 (4.011)
One symptom	13.6 (151)	14.0 (174)	14.5 (471)	14.2 (796)
Two or more	17.2 (191)	17.1 (213)	12.3 (401)	14.3 (805)
Long-term illness,7th grade				
No long-term illness	69.1 (767)	71.4 (888)	72.8 (2.370)	71.7 (4.025)
Long-term illness	12.9 (143)	12.9 (160)	12.5 (409)	12.7 (712)
Medicine prescribed	18.0 (200)	15.7 (196)	14.7 (478)	15.6 (874)
Long-term illness, 9th grade				
No long-term illness	58 (639)	63 (778)	60 (1.958)	60 (3.375)
Long-term illness	14 (159)	14 (174)	16 (507)	15 (840)
Medicine prescribed	28 (312)	23 (292)	24 (792)	25 (1.396)
Self-rated health, 7th grade				
Good	86.7 (963)	84.6 (1.051)	91.2 (2.968)	88.8 (4.982)
Average or poor	13.3 (148)	15.4 (191)	8.8 (288)	11.2 (627)
Self-rated health, 9th grade				
Good	78.7 (874)	81.6 (1.014)	87.6 (2.851)	84.5 (4.739)
Average or poor	21.3 (237)	18.4 (228)	12.4 (405)	15.5 (870)
Gende				
Girl	34.8 (387)	45.7 (569)	57.0 (1.857)	50.1 (2.813)
Boy	65.2 (724)	54.3 (675)	43 (1.401)	49.88 (2.800)
Immigrant background				
Native	94.5 (1.050)	92.9 (1.155)	93.4 (3.044)	93.5 (5.249)
Immigrant	5.5 (61)	7.2 (89)	6.6 (214)	6.5 (364)
Parental employment				
Both parents working	84.7 (914)	88.4 (1.078)	91.1 (2.946)	89.3 (4.938)
Other	15.3 (165)	11.6 (141)	8.9 (288)	10.7 (594)
Parental education				
High	43.0 (478)	65.8 (818)	82.4 (2.684)	70.9 (3.980)
Low	57.0 (633)	34.2 (426)	17.6 (574)	29.1 (1.633)
Family structure				
Nuclear family	53.2 (591)	62.1 (772)	73.9 (2.407)	67.2 (3.770)
Other	46.8 (520)	37.9 (472)	26.1 (851)	32.8 (1.843)
School performance				
High	1.1 (12)	10.0 (124)	49.7 (1.618)	31.3 (1.754)
Medium	28.9 (321)	56.2 (699)	47.3 (1.542)	45.7 (2.562)
Low	70.0 (776)	33.8 (420)	3.0 (98)	23.1 (1.294)

*Note. SDQ* Strengths and Difficulties Questionnaire

The results of the multilevel multinomial logistic regression analyses are presented in Tables 2, 3 and 4. The differences between schools accounted for 0.64 variance which translates into an intra-class correlation [40] of 16.2%.

**Table 2:** Associations of Educational Aspirations with Health, Sociodemographic Background, and Academic Achievement in the 7th Grade: Multilevel Multinomial Logistic Regression. Odds Ratio (OR) and 95% Confidence Intervals (CI) are Presented

	Health factors (*n* = 5.600)		+Sociodemographics (*n* = 5.525)		+School performance (*n* = 5.522)	
*Applied to academic track is the reference category*	*Vocational track*	*Both tracks*	*Vocational track*	*Both tracks*	*Vocational track*	*Both tracks*
	OR (CI)	OR (CI)	OR (CI)	OR (CI)	OR (CI)	OR (CI)
SDQ						
Normal	1	1	1	1	1	1
Slightly raised	**1.76 (1.39–2.23)**	**1.57 (1.29–1.98)**	**1.60 (1.24–2.07)**	**1.50 (1.18–1.89)**	**1.37 (> 1.00–1.88)**	**1.34 (1.04–1.74)**
High difficulty score	**2.92 (2.05–4.17)**	**2.07 (1.45–2.97).**	**2.50 (1.70–3.67)**	**1.94 (1.34–2.79)**	1.47 (0.91–2.36)	1.43 (0.94–2.15)
Daily health complaints						
No symptoms	1	1	1	1	1	1
One symptom	**1.35 (1.09–1.68)**	**1.29 (1.05–1.59)**	**1.32 (1.04–1.66)**	**1.28 (1.04–1.58)**	**1.36 (1.02–1.81)**	**1.30 (1.03–1.64)**
Two or more	1.20 (0.91–1.58)	1.18 (0.92–1.53)	1.14 (0.85–1.53)	1.15 (0.89–1.50)	1.17 (0.82–1.67)	1.11 (0.83–1.48)
Long-term illness						
No long-term illness	1	1	1	1	1	1
Long-term illness	1.01 (0.81–1.26)	0.97 (0.78–1.19)	1.04 (0.82–1.31)	0.97 (0.78–1.20)	1.22 (0.91–1.63)	1.04 (0.82–1.31)
Medicine prescribed	**1.22 (> 1.00–1.49)**	1.04 (0.86–1.27)	1.19 (0.96–1.47)	1.02 (0.84–1.25)	0.97 (0.74–1.27)	0.94 (0.75–1.18)
Self-rated health						
Good	1	1	1	1	1	1
Average or poor	1.20 (0.95–1.52)	**1.51 (1.22–1.88)**	1.03 (0–80-1.33)	**1.41 (1.14–1.77)**	1.17 (0.86–1.59)	**1.57 (1.23–2.01)**
Gender						
Girl	1	1	1	1	1	1
Boy	**2.82 (2.42–3.29)**	**1.76 (1.53–2.03)**	**3.08 (2.61–3.62)**	**1.81 (1.57–2.09)**	**1.98 (1.61–2.42)**	**1.35 (1.15–1.59)**
Immigrant background						
Native			1	1	1	1
Immigrant			0.88 (0.63–1.24)	1.21 (0.91–1-60)	0.85 (0.56–1.29)	1.22 (0.89–1.68)
Parental employment						
Both parents working			1	1	1	1
Other			**1.24 (0.98–1.58)**	1.04 (0.83–1.32)	1.30 (0.96–1.76)	1.06 (0.82–1.38)
Parental education						
High			1	1	1	1
Low			**5.27 (4.47–6.21)**	**2.07 (1.77–2.43)**	**3.26 (2.66–4.00)**	**1.48 (1.24–1.77)**
Family structure						
Nuclear family			1	1	1	1
Other			**1.99 (1.69–2.35)**	**1.53 (1.32–1.79)**	**1.60 (1.30–1.96)**	**1.29 (1.09–1.52)**
School performance						
High					**0.05 (0.03–0.08)**	**0.18 (0.15–0.23)**
Medium					1	1
Low					**35.15 (27.07–45.65)**	**9.53 (7.40–12.27)**
AIC / BIC	10.194.5 / 10.320.5		9483.0 / 9661.7		7454.9 / 7660.0	

*Notes.* Statistically significant associations are marked bold. Strengths and Difficulties Questionnaire = SDQ. Akaike (AIC) and Bayesian (BIC) information criteria are shown

**Table 3:** Associations of Educational Aspirations with Health, Sociodemographic Background, and Academic Achievement in the 9th Grade: Multilevel Multinomial Logistic Regression

	Health factors (*n* = 5.600)		+Sociodemographics (*n* = 5.525)		+School performance (*n* = 5.522)	
*Applied for academic track is the reference category*	*Vocational track*	*Both tracks*	*Vocational track*	*Both tracks*	*Vocational track*	*Both tracks*
	OR (CI)	OR (CI)	OR (CI)	OR (CI)	OR (CI)	OR (CI)
SDQ						
Normal	1	1	1	1	1	1
Slightly raised	**1.94 (1.58–238)**	**1.76 (1.45–2.14)**	**1.86 (1.50–2.31)**	**1.69 (1.38–2.06)**	**1.56 (1.20–2.04)**	**1.44 (1.16–1.79)**
High difficulty score	**3.08 (2.42–3.91)**	**2.41 (1.89–3.06)**	**2.49 (1.93–3.23**	**2.15 (1.68–2.75)**	**1.59 (1.16–2.19)**	**1.54 (1.17–2.02)**
Daily health complaints						
No symptoms	1	1	1	1	1	1
One symptom	1.08 (0.87–1.22)	1.07 (0.87–1.31)	1.04 (0.82–1.32)	1.08 (0.88–1.33)	1.12 (0.84–1.50)	1.13 (0.89–1.42)
Two or more	**1.45 (1.19–1.76)**	1.22 (0.98–1.52)	1.07 (0.84–1.38)	1.22 (0.98–1.53)	1.03 (0.76–1.40)	1.19 (0.93–1.52)
Long-term illness						
No long-term illness	1	1	1	1	1	1
Long-term illness	0.90 (0.73–1.12)	**0.81 (0.66–0.99**	0.90 (0.72–1.13)	0.83 (0.67–1.02)	0.99 (0.74–1.31)	0.89 (0.71–1.12)
Medicine prescribed	1.03 (0.86–1.23**)**	**0.82 (0.69–0.98)**	1.03 (0.85–1.24)	**0.83 (0.69–0.98)**	1.07 (0.85–1.36)	0.84 (0.69–1.02)
Self-rated health						
Good	1	1	1	1	1	1
Average or poor	**1.45 (1.19–1.76)**	**1.27 (1.04–1-54)**	**1.35 (1.10–1.67)**	1.20 (0.98–1.46)	**1.54 (1.18–2.00)**	**1.29 (1.03–1.61)**
Gender						
Girl	1	1	1	1	1	1
Boy	**2.82 (2.42–3.30)**	**1.78 (1.54–2.05)**	**3.04 (2.58–3.59)**	**1.83 (1.58–2.12)**	**1.97 (1.60–2.42)**	**1.37 (1.17–1.61)**
Immigrant background						
Native			1	1	1	1
Immigrant			0.92 (0.65–1.30)	1.24 (0.94–1.65)	0.87 (0.57–1.32)	1.24 (0.90–1.70)
Parental employment						
Both parents working			1	1	1	1
Other			**1.30 (1.02–1.65)**	1.07 (0.85–1.35)	1.32 (0.98–1.79)	1.07 (0.83–1.39)
Parental education						
High			1	1	1	1
Low			**5.11 (4.22–6.02)**	**2.03 (1.73–2.38)**	**3.21 (2.62–3.95)**	**1.47 (1.23–1.76)**
Family structure						
Nuclear family			1	1	1	1
Other			**1.92 (1.63–2.26)**	**1.53 (1.31–1.78)**	**1.55 (1.27–1.91)**	**1.29 (1.09–1.53)**
School performance						
High					**0.05 (0.03–0.08)**	**0.19 (0.15–0.24)**
Medium					1	1
Low					**35.10 (27.02–45.60)**	**9.49 (7.37–12.23)**
AIC / BIC	10.109.6 / 10.235.6		9431.2 / 9609.9		7442.5 / 7647.6	

*Notes.* Odds Ratio (OR) and 95% Confidence intervals (CI) are presented. Statistically significant associations are marked bold. Strengths and Difficulties Questionnaire = SDQ. Akaike (AIC) and Bayesian (BIC) information criteria are shown

**Table 4:** Associations between Educational Aspirations and Change in Health from the 7th–9th Grade, with Health Factors in the 7th Grade Included in the Analysis to Account for Starting Levels and Potential Ceiling Effects (not Shown): Multilevel Multinomial Logistic Regression. Odds Ratio (OR) and 95% Confidence Intervals (CI) are Presented

	Health factors (*n* = 5.600)^1^		+Sociodemographics (*n* = 5.525)^1,2^		+School performance (*n* = 5.522)^1,2,3^	
*Applied for academic track is the reference category*	*Vocational track*	*Both tracks*	*Vocational track*	*Both tracks*	*Vocational track*	*Both tracks*
	OR (CI)	OR (CI)	OR (CI)	OR (CI)	OR (CI)	OR (CI)
SDQ						
Improved	**0.66 (0.45–0.95)**	0.77 (0.54–1.10)	0.86 (0.57–1.27)	0.86 (0.60–1.25)	1.03 (0.63–1.67)	0.94 (0.62–1.42)
Stable	1	1	1	1	1	1
Worse	**2.11 (1.75–2.54)**	**1.82 (1.52–2.19)**	**2.02 (1.66–2.27)**	**1.76 (1.46–2.12)**	**1.62 (1.27–2.07)**	**1.45 (1.18–1.78)**
Daily health complaints						
Improved	1.04 (0.76–1.42)	1.04 (0.78–1.40)	1.04 (0.74–1.44)	1.00 (0.74–1.34)	1.12 (0.75–1.68)	1.10 (0.79–1.53)
Stable	1	1	1	1	1	1
Worse	1.11 (0.91–1.36)	1.12 (0.93–1.35)	1.04 (0.84–1.29)	1.10 (0.80–1.52)	1.10 (0.84–1.44)	1.16 (0.94–1.44)
Long-term illness						
Improved	1.09 (0.82–1.45)	1.01 (0.77–1.33)	1.08 (0.80–1.46)	0.98 (0.74–1.30)	0.91 (0.62–1.33)	0.87 (0.64–1.19)
Stable	1	1	1	1	1	1
Worse	0.98 (0.82–1.18)	**0.78 (0.65–0.93)**	0.98 (0.81–1.19)	**0.77 (0.65–0.93)**	0.98 (0.77–1.25)	**0.77 (0.63–0.94)**
Self-rated health						
Improved	0.90 (0.59–1.39)	1.04 (0.70–1.54)	0.82 (0.52–1.30)	1.03 (0.69–1.53)	0.79 (0.45–1.38)	0.97 (0.63–1.52)
Stable	1	1	1	1	1	1
Worse	**1.48 (1.17–1.87)**	1.21 (0.95–1.53)	**1.38 (1.08–1.77)**	1.14 (0.89–1.45)	**1.57 (1.15–2.14)**	1.19 (0.91–1.56)
AIC / BIC	10.100.9 / 10.333.0			9708.4/ 9708.4		7753.4/ 7753.4

*Notes.* Analyses were adjusted for ^1^gender, ^2^immigrant background, parental employment, and education, and family structure, and ^3^school performance. Statistically significant associations are marked bold. Strengths and Difficulties Questionnaire = SDQ. Akaike (AIC) and Bayesian (BIC) information criteria are shown

### Health in the 7th grade

Already in 12–13 year-olds (the 7th grade), all health factors were associated with students’ educational aspirations recorded more than 2 years later (Table 2). Concerning the SDQ, having a slightly raised or a high difficulty score, compared to having normal levels of psychosocial problems, was associated with a proportionally higher likelihood to apply for the vocational rather than the academic track. Students with psychosocial problems were also more likely to be undecided about their plans for upper secondary education (i.e., applied for both tracks). The associations were robust to accounting for both sociodemographic background and academic achievement. There was, however, one exception: The associations became non-significant for the high difficulty score category. Having one psychosomatic symptom nearly every day, compared no daily health complaints, was associated with applying for the vocational track. Also, students who were undecided in their future educational plans were more likely to report one psychosomatic symptom. Both associations remained significant after including sociodemographic background variables and academic achievement in the model. Having medicine prescribed by the doctor, compared to no long-term illness, was positively associated with applying for the vocational track. In the models that controlled for all other predictors, having a long-term illness was no longer significantly associated with educational aspirations. Students who reported average or poor health, compared to good health, were more likely to belong to the group that had not decided yet and had thus applied for both educational tracks and these associations were robust to controlling for sociodemographic background and academic achievement.

### Health in the 9th grade

We also found significant associations when health was assessed in the same year (in the 9th grade, at the age of 15–16 years) in which Finnish students have to decide about their upper secondary education (Table 3). The more psychosocial problems were reported, the more likely the adolescents were to apply for the vocational track instead of the academic track or the more often they were undecided in their choice between tracks. Similar to the results for the SDQ in the 7th grade, all associations persisted over and above sociodemographic background and academic achievement. There was also a weak association between daily health complaints and educational aspirations in this age group. Having two or more psychosomatic symptoms nearly every day, compared to no symptoms, was related to applying for the vocational track rather than the academic track. Having a long-term illness with and without medicine prescribed was associated with being less undecided. The association for the use of medicine persisted over and above sociodemographic control variables. Educational aspirations were also robustly associated with self-rated health with one exception: reporting average or poor health, compared to good health, was no longer associated with applying for both educational tracks when including sociodemographic background into the model.

### Socio-demographic background and academic achievement

Of the sociodemographic variables, all but immigrant background showed associations with students’ future plans for upper secondary education (Tables 2 and 3). Across the models, boys were less likely than girls to apply for academic track only. Applying for the academic track, instead of the vocational track or to both, was associated with students’ parental background in terms of higher education and nuclear family structure, while the association between applying for vocational track and parental employment disappeared after accounting for academic achievement. Unsurprisingly, especially academic achievement was a very strong and significant predictor of educational aspirations.

### Changes in health from the 7th to the 9th grade

The models that used the data of both surveys simultaneously to assess within-person change from the 7th to the 9th grade and its association with educational aspirations are presented in Table 4. We found a relationship of worsening of health in regards to the SDQ with applying for the vocational track and applying for both tracks. Improvement in this health factor, however, was only weakly associated with applying for the vocational track. Moreover, getting worse health in regards to long-term illness was associated with a decreased likelihood of applying for both educational tracks. Finally, worsening self-reported health over time increased the likelihood to apply for the vocational track. Remarkably, all associations between educational aspirations and increasingly worse health persisted when the sociodemographic background and academic achievement were controlled for.

## Discussion

Educational aspirations, measured by applying for academic versus vocational track or both, were associated with all studied health factors at the age of 12–13 as well the age of 15–16 years. Most associations remained significant after controlling for students’ sociodemographic background and academic achievement. Our results support the health selection hypothesis, i.e. poor health leads to lower educational attainment; students with better health in adolescence were more likely to apply for the academic track, and those who were less healthy were more likely to apply for the vocational track. In line with our expectations, health in the group of undecided students who had applied for both educational tracks lay in between.

In our data, lower educational aspirations were related to having psychosocial problems assessed with the SDQ, daily health complaints assessed with the frequency of psychosomatic symptoms, and average or poor self-rated health. Previous studies did not provide a clear picture of whether poor health distorts educational aspirations [23, 24] and also associations between health and educational attainment in adolescence have been found to be mixed [6, 9–16].

The associations of the SDQ, daily health complaints, and self-rated health with students’ educational aspirations stayed but attenuated after controlling for sociodemographic background and academic achievement which supports the independent effect of health factors in the creation of socioeconomic health inequalities. The plausibility of the health selection hypothesis was further strengthened by the finding that the group of those students whose health worsened over time in terms of the SDQ and self-rated health, had on average lower academic aspirations. This makes it less likely that an unobserved third factor that influences both health and educational aspirations had confounded the observed associations. Previous studies that were able to examine fixed effects estimates, similarly, found that the education-health gradient is largely shaped by health selection [6, 11].

With this study we wanted to find out whether students’ plans after compulsory schooling are already patterned by their health in the 7th grade (age 12–13) when students start lower secondary education or whether health matters only at the end of the 9th grade (age 15–16 years) at the time when they apply to upper secondary education. On average, the effect of health was weaker at age 12–13 than at age 15–16. As the differences fall within the respective CIs, however, these associations do not seem to be significantly modified by being assessed in the 7th or 9th grade. Thus, both times seem to be crucial for determining students’ successful educational paths into adulthood. At the same time, the results indicate that health in adolescence influences students’ future plans even if assessed years before the choice between academic and vocational track has to be made in Finland. This finding aligns well with research on the influence of health disadvantage in early childhood on later educational attainment [4] and shows the importance of adolescence as a formative period of life.

Inconsistent results were observed for long-term illness, which related to lower educational aspirations when being assessed in the 7th grade but instead to higher educational aspirations when being assessed in the 9th grade. Adolescents that reported worsening of health between the measurement points in regards to long-term illness also applied proportionally less often for both educational tracks instead of the academic track only. That the associations had the opposite sign at different ages matches the mixed results obtained in previous work on adolescents with long-term illness and educational attainment [9, 10, 15]. Our results further show that health-related selection may work differently for different health factors [13].

The significant proportion of the variance attributable to differences between schools suggests that the role of student composition and contextual factors cannot be ignored in the complex relationship between health and educational aspirations [34, 41].

As expected, students’ educational aspirations were related to their parents’ education and employment as well as their academic achievement. Both this result and the fact that educational aspirations and health in adolescence showed an association over and above students’ academic achievement might point to the bidirectional nature of the relationships [4, 12, 42]. Health and academic achievement are most likely interconnected since performance at school already reflects students’ earlier health, and perceptions related to academic success and failure are probably intertwining with health perceptions over the school years [11, 34, 43]. It is also well known that even in the Finnish welfare state social factors of the family influence students’ educational choices and trajectories [25–27, 29]. Thus, the interplay between the mechanisms of health selection and social causation in the production of health inequality was visible in our data which highlights that they can have different influences at different periods of life course [5, 8].

### Limitations and strengths

We cannot exclude bias in our results due to selective attrition. Without attrition, however, the observed effects of the studied health and social factors on educational aspirations might have been even stronger because those who were less healthy and from a more disadvantaged family background were less likely to participate in the second survey.

Among the considerable strengths of the research is the fact that we used a longitudinal multilevel design to understand how health in adolescence links to the choice between educational tracks that took into account the significant effect of the school attended on educational aspirations. Very few, if any other large adolescent cohorts have covered health and education as comprehensively both in terms of health indicators and the opportunity to follow the same individuals over the transition to further education after the compulsory schooling ends. Assessing health longitudinally enabled us to identify those periods in adolescence which are sensitive for their successful paths into adulthood and to examine the effects of within-person change in health over time. Educational aspirations were assessed objectively by obtaining from the national registry covering all students in the country, the choices they have made when applying to upper secondary education. Using national registry data further reduced measurement error and the amount of missing data due to nonresponse.

## Conclusions

Our findings show that selection by health factors into different educational trajectories takes place already at the early teenage much before the adolescents need to choose which educational track – if any – they wish to apply after the compulsory schooling. Our findings support the health selection hypothesis in the creation of health inequalities: those whose health is worse, more often had lower educational aspirations than those whose health is better. That health factors had an effect over and above sociodemographic background, and school performance shows that health in adolescence is independently associated with the plans of students for their further education. Our findings also show the importance of adolescence in the creation of inequalities. More studies are needed to reveal which measures would be effective in helping students with poor health to achieve their full educational potential.

## Supplementary information

**Additional file 1: Table S1.** Change in Health from the 7th–9th Grade, % (n).

## Data Availability

The access for data for external researchers may be granted based on a written request to AR and RH including a research plan and a data management plan. AR is responsible for the health part of the school data. RH is responsible for the education part of the school data.

## Abbreviations

AIC: Akaike information criteria
BIC: Bayesian information criteria
CI: Confidence intervals
GPA: Grade point average
MetLoFin: Metropolitan Longitudinal Finland
OR: Odds ratios
SDQ: Strengths and Difficulties Questionnaire
TENK: Finnish National Board on Research Integrity
