# Supplementary material for: Health and educational aspirations in adolescence: a longitudinal study in Finland
Source: BMC Public Health. 2019 Nov 4;19:1447. doi: 10.1186/s12889-019-7824-8 (PMC6829805; doi:10.1186/s12889-019-7824-8)
Supplement: Supplementary file 1 — Additional file 1: Table S1. Change in Health from the 7th–9th Grade, % (n). [file 12889_2019_7824_MOESM1_ESM.docx]

Supplement to Dobewall, H. et al. “**Health and educational aspirations in adolescence: A longitudinal study in Finland**” BMC Public Health

Table S1. Change in Health from the 7^th^-9^th^ Grade, % (n)

| SDQ, change 7th-9th grade | | |
| --- | --- | --- |
|  | Improved | 7.2. (403) |
|  | Stable | 73.0 (4.098) |
|  | Worse | 19.8 (1.113) |
| Daily health complaints, change 7th-9th grade | | |
|  | Improved | 12.5 (700) |
|  | Stable | 68.4 (3.841) |
|  | Worse | 19.1 (1.073) |
| Long-term illness, change 7th-9th grade | | |
|  | Improved | 10.0 (560) |
|  | Stable | 66.9 (3.757) |
|  | Worse | 23.1 (1.297) |
| Self-rated health, change 7th-9th grade | | |
|  | Improved | 5.8 (328) |
|  | Stable | 83.9 (4711) |
|  | Worse | 10.2 (575) |
